# Differential Modulation of the European Sea Bass Gut Microbiota by Distinct Insect Meals

**DOI:** 10.3389/fmicb.2022.831034

**Published:** 2022-04-12

**Authors:** Fábio Rangel, Paula Enes, Laura Gasco, Francesco Gai, Bela Hausmann, David Berry, Aires Oliva-Teles, Claudia R. Serra, Fátima C. Pereira

**Affiliations:** ^1^Department of Biology, Faculty of Sciences, University of Porto, Porto, Portugal; ^2^CIMAR/CIIMAR Interdisciplinary Centre of Marine and Environmental Research, University of Porto, Matosinhos, Portugal; ^3^Department of Agricultural, Forest and Food Sciences, University of Turin, Torino, Italy; ^4^Institute of Science of Food Production, National Research Council, Torino, Italy; ^5^Joint Microbiome Facility of the Medical University of Vienna and the University of Vienna, Vienna, Austria; ^6^Department of Laboratory Medicine, Medical University of Vienna, Vienna, Austria; ^7^Division of Microbial Ecology, Department of Microbiology and Ecosystem Science, Centre for Microbiology and Environmental Systems Science, University of Vienna, Vienna, Austria

**Keywords:** exuviae, *Bacillus*, *Paenibacillus*, *Dicentrarchus labrax*, chitin, *Hermetia illucens*, *Tenebrio molitor*, feedstuff

## Abstract

The aquaculture industry is one of the fastest-growing sectors in animal food production. However, farming of carnivorous fish strongly relies on the use of wild fish-based meals, a practice that is environmentally and economically unsustainable. Insect-based diets constitute a strong candidate for fishmeal substitution, due to their high nutritional value and low environmental footprint. Nevertheless, data on the impact of insect meal (IM) on the gut microbiome of farmed fish are so far inconclusive, and very scarce in what concerns modulation of microbial-mediated functions. Here we use high-throughput 16S rRNA gene amplicon sequencing and quantitative PCR to evaluate the impact of different IMs on the composition and chitinolytic potential of the European sea bass gut digesta- and mucosa-associated communities. Our results show that insect-based diets of distinct origins differently impact the gut microbiota of the European sea bass (*Dicentrarchus labrax*). We detected clear modulatory effects of IM on the gut microbiota, which were more pronounced in the digesta, where communities differed considerably among the diets tested. Major community shifts were associated with the use of black soldier fly larvae (*Hermetia illucens*, HM) and pupal exuviae (HEM) feeds and were characterized by an increase in the relative abundance of the Firmicutes families *Bacillaceae*, *Enterococcaceae*, and *Lachnospiraceae* and the Actinobacteria family *Actinomycetaceae*, which all include taxa considered beneficial for fish health. Modulation of the digesta community by HEM was characterized by a sharp increase in *Paenibacillus* and a decrease of several Gammaproteobacteria and Bacteroidota members. In turn, a mealworm larvae-based diet (*Tenebrio molitor*, TM) had only a modest impact on microbiota composition. Further, using quantitative PCR, we demonstrate that shifts induced by HEM were accompanied by an increase in copy number of chitinase ChiA-encoding genes, predominantly originating from *Paenibacillus* species with effective chitinolytic activity. Our study reveals an HEM-driven increase in chitin-degrading taxa and associated chitinolytic activity, uncovering potential benefits of adopting exuviae-supplemented diets, a waste product of insect rearing, as a functional ingredient.

## Introduction

Human demand for fish protein has led to a large expansion of the aquaculture industry. The production of carnivorous species is heavily reliant on the use of fish meal (FM) as a major protein source for aquafeeds (Hoffmann et al., 2020). The reduced availability of FM and its subsequent increase in price due to depletion of wild fish stocks, together with the environmental impacts associated with FM use, is driving the sector to search for new sustainable feedstuffs (Gasco et al., 2020a).

Recently, insects started to be tested as a novel animal protein and lipid source with the potential to be consumed by humans, livestock, and fish (van Huis et al., 2013; Tomberlin and van Huis, 2020; van Huis, 2020). In Europe, interest in insect meal (IM) sparked upon implementation of a European Union (EU) Directive (Regulation No 2017/893), effective after July 2017, which authorized the use of seven insect species in aquafeeds. The nutritional value of insects can greatly vary depending on species, development stage, or processing method. Overall, IM protein content can vary between 30% and 68% dry matter (DM), having a more balanced amino acid profile than plant feedstuffs, and providing higher levels of essential amino acids (Nogales-Mérida et al., 2018; Gasco et al., 2020a). Lipid levels usually fluctuate between 10% and 30% DM (Nogales-Mérida et al., 2018; Gasco et al., 2020a), but can be reduced to 9%–5% DM by defatting processes. Moreover, depending on the species and rearing material, IM presents a variable fatty acid profile, which can be modulated through the diets of the insects to meet the needs of a particular fish species (Barroso et al., 2014; Liland et al., 2017; Nogales-Mérida et al., 2018; Danieli et al., 2019; Benzertiha et al., 2020; Gasco et al., 2020b; Hoc et al., 2021; Pulido et al., 2022).

From the seven insect species authorized for use in aquafeeds in the EU, *Hermetia illucens* (HI) and *Tenebrio molitor* (TM) are among the most investigated (Gasco et al., 2019). Both are commonly used in their larval stage, characterized by a protein-rich content, and suitable amino acid and fatty acid profiles for inclusion in aquafeeds (Hawkey et al., 2021). Additionally, during the different life cycle stages, these insects go through metamorphosis, leaving behind chitin and protein-rich exoskeletons or shells, known as exuviae, an untapped valuable waste for fish feeds (Purkayastha and Sarkar, 2020). Data on fish growth performance are generally similar between diets containing HI or TM, with positive outcomes obtained when the IM inclusion levels are below 30% of the total meal (Magalhães et al., 2017; Terova et al., 2019; Caimi et al., 2020, 2021; Hoffmann et al., 2020; Stejskal et al., 2020; Shafique et al., 2021). Dietary HI or TM inclusions over 30% generally lead to detrimental effects in growth performance and are associated with decreases in the apparent digestibility coefficients of crude protein and/or lipids (Piccolo et al., 2017; Guerreiro et al., 2021). The lower digestibility of these ingredients has been proposed to be correlated with the increased dietary chitin content, a hypothesis supported by *in vitro* results (Marono et al., 2015) and *in vivo* trials (Piccolo et al., 2017; Mastoraki et al., 2020; Guerreiro et al., 2021). Chitin, through a process called sclerotization, can bind and entrap proteins and lipids in a structural matrix, such as the one that makes up the exoskeleton of Arthropoda (Resh and Cardé, 2009). This leads to reduced access of proteases and lipases to their substrates, leading to a lower IM digestibility (De Marco et al., 2015). This has been verified in digestibility trials with IM-containing diets (Piccolo et al., 2017; Chemello et al., 2020; Guerreiro et al., 2021). Therefore, for more efficient use of IM, chitinolytic activity is required. Endogenous chitinolytic activity has been detected in the digestive system of some marine fish such as juvenile cobia (*Rachycentron canadum*) and cod (*Gadus morhua*; Danulat and Kausch, 1984; Fines and Holt, 2010), although it was found to be lacking in others including turbot (*Psetta maxima*) and meagre (*Argyrosomus regius*; Kroeckel et al., 2012; Guerreiro et al., 2021). In European sea bass (*Dicentrarchus labrax*), although chitinase-encoding genes have been found (Calduch-Giner et al., 2016), to the best of the authors knowledge, no chitinolytic activity measurements have been made.

The gastrointestinal (GI) tract, however, depends not only on its endogenous enzymes for the digestive process. Fish and other animals rely on symbiotic relationships with their GI tract microbial community, which through enzymatic secretion, can breakdown specific, otherwise indigestible dietary components, such as chitin, and thereby help hosts to obtain energy from inaccessible nutrient sources (Roeselers et al., 2011; Ray et al., 2012; Tremaroli and Bäckhed, 2012). Dietary changes create different nutrient niches by promoting the use of novel substrates by different microbes (Ringø et al., 2015; Serra et al., 2021). Such alterations in fish gut microbiomes can alter the microbial digestive enzymatic contribution, and ultimately, lead to a better use of novel ingredients (Gasco et al., 2021).

Understanding the main modifications of fish gut microbiota due to dietary composition, as well as microbiota’s role in digestion and host fitness, is important for the successful introduction of novel ingredients into aquafeeds. However, regarding IM in fish diets, data on the species-specific gut microbiota responses to chitin-containing diets and their mediated processes are still scarce, or non-existent for the case of exuviae diets (Perry et al., 2020). As such, this study aimed to assess the effect of three different IM, namely, HI and TM larvae meals, and HI exuviae meal (HEM), on European sea bass gut microbiota using Illumina MiSeq 16S rRNA gene amplicon sequencing. To help elucidate the impact of chitin on the gut microbiota, a control FM-based diet supplemented with commercial chitin was also included. As previous studies have reported different responses of digesta- and mucosa-associated gut communities to dietary changes (Serra et al., 2021), we have here analyzed the impact of IM in these two intestinal sites separately. Additionally, using quantitative PCR targeting bacterial chitinase genes, we aimed to unveil the potential chitinolytic contribution of the microbiota under different diets.

## Materials and Methods

### Experimental Diets and Proximate Analysis

Five isoproteic (45%) and isolipidic (18%) diets were formulated: a control diet (CTR); a diet similar to the CTR with 5% commercial chitin (Sigma-Aldrich Química, S.L., Sintra, Portugal) supplementation (CHIT5); and three other diets with 25% inclusion of partially defatted black soldier fly (*Hermetia illucens*) larvae meal (HM25); 25% inclusion of partially defatted yellow mealworm (*Tenebrio molitor*) larvae meal (TM25); and 25% inclusion of *H. illucens* exuviae meal (HEM25), respectively. The inclusion of the HM (crude protein: 60.3% dry matter (DM); gross lipid: 6.4% DM; chitin 6.8% DM), TM (crude protein: 69.5% DM; gross lipid: 14.1% DM; chitin 5.2% DM) and HEM (crude protein: 64.3% DM; gross lipid: 8.1% DM; chitin 7.2% DM) corresponded to a 39%, 51%, and 46% of FM replacement, respectively. Diets were supplemented with monoammonium phosphate to avoid phosphorus imbalance. All dietary ingredients were finely ground, well mixed, and dry pelleted in a laboratory pellet mill (California Pellet Mill, CPM Crawfordsville, IN, United States) through a 2 mm die. The pellets were then dried in an oven at 40°C for 24 h and stored at −20°C in airtight bags until used. Ingredients and proximate composition of the experimental diets are presented in Supplementary Table 1. Chemical analysis of the ingredients and experimental diets (dry matter, protein, lipid, and ash) was done following the Association of Official Analytical Chemists methods (AOAC, 2000). HM, TM, HEM and the final diets formulated with these meals were analyzed for chitin composition according to Guerreiro et al. (2020).

### Animals and Experimental Conditions

The trial was directed by accredited scientists (following FELASA category C recommendations) and conducted according to the European Union Directive (2010/63/EU) on the protection of animals for scientific purposes. European sea bass (*Dicentrarchus labrax*) were obtained from Sonríonansa (Cantabria, España), and transported to the Marine Zoology Station (Porto University, Porto, Portugal), where the experiment was conducted. After transport, fish were submitted to a quarantine period of 4 weeks. During this period, fish were fed a commercial diet (18% lipids and 44% protein, Aquasoja Sustainable Feed; Sorgal, Ovar, Portugal). Thereafter, 15 groups of 15 fish with an initial mean body weight of 53.7 ± 2.67 g were established. Experimental diets were randomly assigned to triplicate tanks. The trial was conducted in a recirculating aquaculture system equipped with 15 fiberglass tanks of 100 L water capacity, thermo-regulated to 22.7 ± 0.3°C and supplied with a continuous flow of seawater (36.0 ± 0.5 g L^−1^ salinity, *circa* 7 mg L^−1^ oxygen). The photoperiod was set, with artificial illumination, to 12:12 h light:dark. The trial lasted 8 weeks, and fish were fed by hand twice a day, 6 days per week, until apparent visual satiation. At the end of the feeding trial, all fish reached 2.1–2.2 times their initial body weight, as determined by randomly weighing one fish per tank.

### Sampling

Two fish per tank were randomly sacrificed with an anesthetic overdose (3.0 ml L^−1^ ethylene glycol monophenyl ether), 4 h after the first meal, to assure presence of digesta in the gut. Whole-gut (without pyloric caeca) was aseptically excised and squeezed to collect the digesta contents. Mucosa samples were obtained by scraping the internal intestinal mucosa after opening the intestines in their longitudinal axis. To overcome inter-fish variation, the resulting material from the two fish was pooled into one sample per tank, immediately frozen in liquid nitrogen and stored at −80°C until further analysis to assess differences between dietary groups.

### DNA Extraction

DNA was extracted from 250 mg of either the collected gut contents or gut mucosa, which were transferred to 2-ml sterile tubes containing 400 mg of 425–600 μm glass beads (Sigma-Aldrich, Germany) and 500 μl of STE buffer. The samples were homogenized in a Precellys homogenizer (Bertin Instruments, France) using 3 cycles of 60 s at 4,500 rpm with 60 s intervals on ice between cycles. The samples were incubated for 15 min at 75°C, centrifuged (16,000 *g* for 1 min at 4°C) and 500 μl of supernatant was collected to a new 2-ml sterile tube. DNA was extracted from the supernatant, using the GES buffer (5 M guanidine thiocyanate, 0.5 M EDTA, 10% N-lauroylsarcosine) and phenol methodology of Pitcher et al. (1989), with the modifications described by Santos et al. (2021). Nucleic acid pellets were finally resuspended in 50 μl of nuclease free water. As a negative control for the extraction procedure, a sample with only the lysis buffer was processed in parallel with all samples.

### Amplification and Sequencing of the 16S rRNA Genes

Amplification of bacterial and archaeal 16S rRNA genes from DNA extracts was performed with a two-step barcoding approach (UDB-H12; Pjevac et al., 2021). In the first-step PCR, the primers 515F (5′-GTGYCAGCMGCCGCGGTAA-3′; Parada et al., 2016) and 806R (5′-GGACTACNVGGGTWTCTAAT-3′; Apprill et al., 2015), including a 5′-head sequence for 2-step PCR barcoding, were used. PCRs, barcoding, library preparation and Illumina MiSeq sequencing were performed by the Joint Microbiome Facility (Vienna, Austria) under project number JMF-2002-2. First-step PCRs were performed in triplicate (12.5 μl vol per reaction) with the following conditions: 1X DreamTaq Buffer (Thermo Fisher), 2 mM MgCl_2_ (Thermo Fisher), 0.2 mM dNTP mix (Thermo Fisher), 0.2 μM of forward and reverse primer each, 0.08 mg ml^−1^ Bovine Serum Albumin (Thermo Fisher), 0.02 U Dream Taq Polymerase (Thermo Fisher), and 0.5 μl of DNA template. Conditions for thermal cycling were: 95°C for 3 min, followed by 30 cycles of 30 s at 95°C, 30 s at 52°C and 50 s at 72°C, and finally 10 min at 72°C. Triplicates were combined for barcoding (with eight PCR cycles). Barcoded samples were purified and normalized over a SequalPrep Normalization Plate Kit (Invitrogen) using a Biomek NXP Span-8 pipetting robot (Beckman Coulter), and pooled and concentrated on PCR purification columns (Analytik Jena). Indexed sequencing libraries were prepared with the Illumina TruSeq Nano Kit as described previously (Herbold et al., 2015), and sequenced in paired-end mode (2× 300 bp; v3 chemistry) on an Illumina MiSeq following the manufacturer’s instructions. The workflow systematically included four negative controls (PCR blanks, i.e., PCR-grade water as template) for each 90 samples sequenced.

### Analysis of 16S rRNA Gene Amplicon Sequences

Amplicon pools were extracted from the raw sequencing data using the FASTQ workflow in BaseSpace (Illumina) with default parameters (Pjevac et al., 2021). Input data were filtered for PhiX contamination with BBDuk^1^ (BBTools, Bushnell B). Demultiplexing was performed with the python package demultiplex (Laros JFJ)^2^ allowing one mismatch for barcodes and two mismatches for linkers and primers. DADA2 R package version 1.16.0^3^ (R 4.0.2; Callahan et al., 2016a) was used for demultiplexing amplicon sequencing variants (ASVs) using a previously described standard protocol (Callahan et al., 2016b). FASTQ reads were trimmed at 150 nt with allowed expected errors of 2. Taxonomy was assigned to 16S rRNA gene sequences based on SILVA taxonomy (release 138) using the DADA2 classifier (Callahan et al., 2016a). ASVs assigned as Chloroplast were considered dietary plant chloroplast contaminants and removed from the analysis. Sequencing of an extraction control yielded only nine reads, part of these classified as a *Pseudomonas* sp. ASV (ASV_lwc_fxo), a common contaminant found in PCR reagents that was therefore also removed from analysis. In addition, we did find five reads belonging to ASV_tnz_8cx (average read abundance across non-control samples: 478 reads) and ASV_hek_q1g (average read abundance across non-control samples: 187 reads). As these ASVs are present in the samples in high abundance, we do believe that very few reads detected in the negative control may not be derived from contaminants (water or others) in reagents, but from the well-known low levels of cross-contamination that can be detected when handling multiple samples in parallel (Minich et al., 2019) and were therefore retained for further analysis. Amplicon sequence libraries were rarefied to 3,100 reads per sample and analyzed using the vegan (v2.5-.6; Oksanen et al., 2020) and phyloseq (v1.30.0; McMurdie and Holmes, 2013) packages of the software R^3^ (R 4.0.2). A total of 740 unique ASVs were retained after filtering and rarefying. Sample coverage, alpha-diversity metrics, Bray–Curtis dissimilarity and non-metric multidimensional scaling (NMDS) were calculated using the phyloseq R package. Ellipses were drawn on NMDS plots using vegan’s veganCovEllipse() function. Rarefaction curves were generated using the vegan’s function rarecurve(). DESeq2 (v1.26.0; Love et al., 2014) implemented in phyloseq was used to determine significant differences in ASV, genera and family abundances between diets and/or intestinal sampling site. Only ASVs that had in total ≥10 reads were considered for comparisons by DESeq2 analyses. All statistical analysis on microbiome data were carried out *via* the software R (R 3.6.2), and statistical tests and value of *p* are indicated in the main text and figure legends.

### Amplification of Chitinase A Genes

Forward (5′-GATATCGACTGGGAGTTCCC-3′) and reverse (5′-CATAGAAGTCGTAGGTCATC-3′) primers previously developed to target the Chitinase gene A (*chiA*) from bacteria of marine environments (Ramaiah et al., 2000) were used to detect and quantify the presence of bacterial ChiA-encoding genes in the digesta and mucosa of the European sea bass, as well as in the genomes of *Paenibacillus* spp. isolates. Initial PCR amplifications to check primer specificity were performed on DNA samples from the digesta of fish fed the HEM25 diet, using the following conditions: 1X DreamTaq Buffer (Thermo Fisher), 2 mM MgCl_2_ (Thermo Fisher), 0.2 mM dNTP mix (Thermo Fisher), 0.2 μM of forward and reverse primer each, 0.08 mg ml^−1^ Bovine Serum Albumin (Thermo Fisher), 0.02 U Dream Taq Polymerase (Thermo Fisher), and 2 μl of DNA template for a total 20 μl reaction volume. Conditions for thermal cycling were: 95°C for 4 min, followed by 30 cycles of 1 min each at 95°C, 55°C, and 72°C, and finally 10 min at 72°C. PCR products were checked on a 0.8% agarose gel for any visible primer dimer or double bands. Triplicates were combined and purified with the innuPREP PCRpure Kit (Analytik Jena). Clean PCR products were pooled and ligated and transformed into vectors using the TOPO® TA Cloning® kit (Thermo Fisher) according to the manufacturer’s protocol. Clones were checked using PCR with the universal M13 forward and reverse primers and the purified PCR product was sent for Sanger sequencing at the company Microsynth Austria GmbH. The sequencing results were analyzed by BLASTX against a database of non-redundant bacterial and archeal protein sequences from NCBI^4^ to check for specificity of the primers.

### Quantitative PCR of *chiA* Gene Copy Number Density

Extracted digesta and mucosa DNA (2 μl) was subjected to quantitative PCR using 0.2 μM of primers specifically targeting bacterial *chiA* (Ramaiah et al., 2000) and 1x SYBR green Master Mix (Bio-Rad) in a total reaction volume of 20 μl. Standard curves were generated from a 416 bp PCR fragment amplified from a clone containing a chitinase sequence 100% identical to a ChiA sequence from *Paenibacillus thiaminolyticus* (NCBI accession number: WP_143798588.1). The purified PCR product was quantified using a Qubit dsDNA BR Assay Kit in a Qubit 4 fluorometer (Thermo Fisher). For standard preparation, a 10-fold dilution series was prepared from 10^8^ to 10^0^ copies per μl and checked for optimal efficiency. Amplification and detection were performed using a CFX96™ Real-Time PCR Detection System (Bio-Rad) using the following cycling conditions: 95°C for 5 min, followed by 40 cycles of 92°C for 1 min, 55°C for 1 min, and 72°C for 1 min. To determine the specificity of PCR reactions, melt curve analysis was carried out after amplification by slow cooling from 95°C to 60°C, with fluorescence collection at 0.3°C intervals and a hold of 10 s at each decrement. Only assays with amplification efficiencies above 85% were considered for analysis.

### Phylogenetic Analysis

A reference tree was constructed using near full-length 16S rRNA gene reference sequences (>1,200 bp) derived from selected isolates using IQ-TREE server (Trifinopoulos et al., 2016) with automatic model selection (TIM3 + F + I + G4) and ultra-fast bootstrapping with 1,000 replicates. Query sequences from ASVs and isolates obtained in this study were then added to the reference tree using the Evolutionary Placement Algorithm of RAxML (Stamatakis, 2014), using the GTRGAMMA model.

### Bacterial Isolation, Identification, and Chitinolytic Assays

*Paenibacillus* spp. were isolated from a 1:5 (wt:vol) dilution of each pooled digesta sample immediately before freezing. Serial dilutions (10^−0^, 10^−1^, 10^−2^, 10^−3^, and 10^−4^) in Bott & Wilson (B&W) salts were heat treated for 20 min at 65°C (Nicholson and Setlow, 1990), spread on Luria Bertani (LB) medium plates and incubated for up to 96 h at room temperature (25 ± 1°C). Colonies with the distinct morphologies were purified and stored at −80°C in 25% glycerol. Chitinolytic activity was evaluated by the presence of a chitin-hydrolysis halo around colonies of each isolate when grown up to 14 days at 28°C in a chemically defined media (w/v, 0.648% Na_2_HPO_4_, 0.3% KH_2_PO_4_, 0.05% NaCl, 1% NH_4_Cl, 0.02% MgSO_4_, 0.01% Yeast extract, 1% colloidal chitin) containing 1.6% (w/v) agar. The *Chromobacterium violaceum* ATCC 12472, known to possess chitinolytic activity, was used as a positive control (Liu et al., 2013). The chromosomal DNA of each isolate was purified from a 2 ml overnight LB culture (37°C, 120 rpm) using the ZymoBIOMICS™ DNA Miniprep Kit (Zymo Research Corp., Irvine, CA, United States), according to the manufacturer instructions. Isolates identification was done by partially sequencing (STABVIDA, Caparica, Portugal) the 16S rRNA gene, previously amplified by PCR with oligonucleotide primers 16S-27F and 16S-1492R (Weisburg et al., 1991), and analyzed by BLAST against the GenBank nonredundant (nr) nucleotide database.^4^

## Results

**Figure 1:**
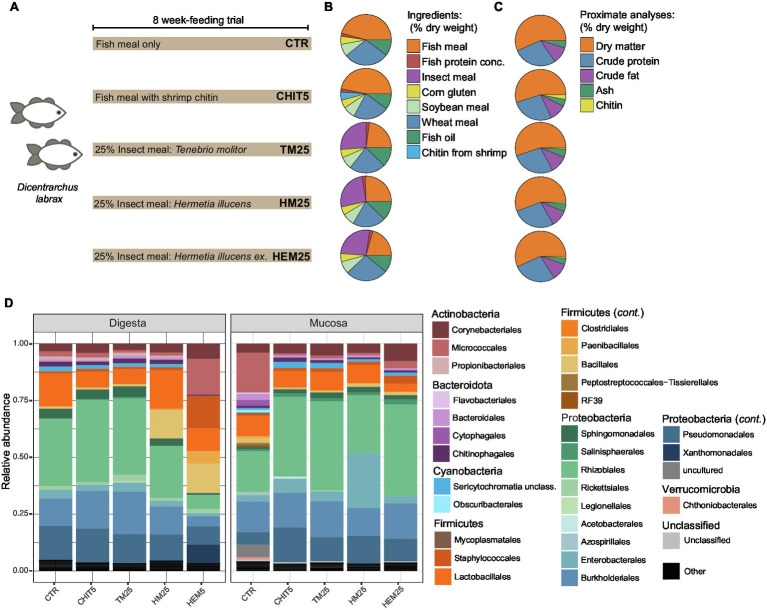
Feeding diets and gut microbiota composition of the European sea bass. **(A)** Three tanks containing 15 *Dicentrarchus labrax* fish each were fed with the five different depicted diets for 8 weeks (see Materials and Methods). *ex*, exuviae. Three different tanks per diet were included in the trial. **(B)** Percentages (dry weight basis) of the different ingredients in each diet. Only major ingredients (final percentage in diet >1.0%; Supplementary Table 1) are shown. **(C)** Despite different compositions, proximate analyses show that all diets contain similar amounts of dry matter, crude protein, crude fat and ash, while levels of chitin are rather more variable (Supplementary Table 1). **(D)** Phylum- and Order-level relative abundance profile of the gut microbiota of digesta and mucosa samples from fish fed the different diets depicted in **(A)**. Orders with <1% relative abundance are collapsed into the category “Others.” Each bar represents the average of three different samples per diet for each habitat, each sample originating from a different tank.

To understand the impact of different IM types on the European sea bass gut microbiota, fish were fed five isoproteic and isolipidic diets, including: a control FM-based diet (CTR), a CTR diet supplemented with 5% of a commercial chitin from shrimp shells (CHIT5), and three diets including 25% of TM or HI larvae meals or HI exuviae meal (diets TM25, HM25 and HEM25, respectively; Figures 1A–C; Supplementary Table 1). Dietary chitin content was similar among these diets (1.3% in TM25, 1.7% in HM25 and 1.8% in HEM25). Digesta (gut content) and mucosa samples (scraping of the mucosa from emptied gut) were collected and DNA was isolated and subjected to Illumina MiSeq sequencing of the V4 region of the 16S rRNA gene. After quality filtering, a total of 311,727 high-quality sequences were retained with an average of 10,390 high-quality sequences per sample. Sequencing of a DNA extraction control sample yielded a very low number of reads (<10 reads), partly belonging to a clear contaminant found in PCR reagents (*Pseudomonas* sp.). This ASV and all ASVs classified as chloroplast were removed from our analysis (see Materials and Methods). After rarefying, the ASV coverage was higher than 0.97 for all sequenced samples (Supplementary Table 2), indicating that sequencing depth was adequate to capture most of the diversity present (Supplementary Figure 2). Overall, the European sea bass gut microbiota was dominated by the phylum Proteobacteria (68.3% in relative abundance), followed by Firmicutes (17.2%), Actinobacteria (8.4%) and Bacteroidota (2.3%; Figure 1D; Supplementary Table 3). Within the phylum Proteobacteria there is a dominance of organisms belonging to the Alphaproteobacteria orders Rhizobiales and Sphingomonadales, and Gammaproteobacteria orders Burkholderiales and Pseudomonadales (Figure 1D). The majority of Firmicutes belong to the order Lactobacillales (Figure 1D). Actinobacteria organisms mostly belonged to the order Corynebacteriales and to the potential pathogenic order Micrococcales.

### Digesta and Mucosa Harbor Distinct Microbial Communities

**Figure 2:**
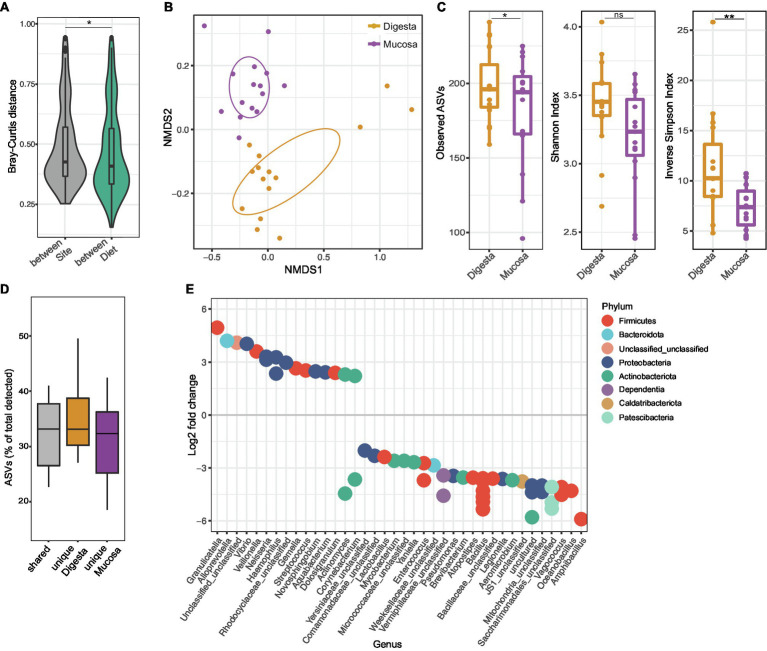
Sampling site as a driver of gut microbial community composition. **(A)** Violin plot representing the calculated Bray–Curtis distances between samples originating from different sampling intestinal sites (grey) or between samples originating from fish fed different diets (green). ^*^p = 0.031; Wilcoxon test. **(B)** Ordination plot based on non-metric multidimensional scaling analysis of Bray–Curtis distances at the ASV level. SD ellipses and point dots representing each sample are depicted and are colored by sampling intestinal site. Stress = 0.057. **(C)** Alpha diversity metrics (number of Observed ASVs, Shannon Index, and Inverse Simpson Index) of the mucosa and digesta samples. Each point represents one sample (*n* = 15 paired mucosa and digesta samples). ^*^p = 0.041; ^**^p = 0.008; ns = non-significant; paired Wilcoxon test. **(D)** Percentage of total ASVs detected in samples originating from the same tank that are shared between the mucosa and corresponding digesta sample, or unique to sampled site. **(E)** Differential abundant ASVs based on DESeq2 analysis (adjusted value of *p* < 0.05, Wald test followed by Benjamini–Hochberg correction for multiple testing) between mucosa and digesta samples (Log2 fold change >2 denotes enrichment in mucosa; Log2 fold change <−2 denotes enrichment in digesta). ASVs are assigned to genus (*x*-axis) and colored by phylum. In **(A,C,D)** boxes represent median, first and third quartile. Whiskers extend to the highest and lowest values that are within one and a half times the interquartile range.

We found that there is a stronger impact of the intestinal site sampled (digesta vs. mucosa) when compared with the impact of the tested diets on the beta-diversity variation of gut communities (Figure 2A; *p* = 0.031, Wilcoxon test). Indeed, the beta-diversity analysis revealed a significant overall impact of the sampled intestinal site on community composition, with microbial communities from digesta and mucosa clustering separately (*p* = 0.004, *r*^2^ = 0.247, PERMANOVA, Figure 2B). Additionally, we found that, on average, only 33% of the total ASVs detected for each pair of digesta and mucosa samples are shared between the two (Figure 2D), although no physical separation exists between them. Digesta communities are more diverse than their mucosal counterparts (Figure 2C; Supplementary Table 4), displaying significantly higher richness (199.2 ± 22.6 vs. 181.3 ± 38.1 observed ASVs, *p* = 0.041, paired Wilcoxon test) as well as a greater Inverse Simpson diversity index (11.4 ± 4.9 vs. 7.5 ± 2.4, *p* = 0.008, paired Wilcoxon test). These results are in line with our analyses that show that higher percentages of total ASVs tend to be uniquely associated with the digesta compared with the mucosa (Figure 2D).

We next sought to investigate which species are preferentially found in each of the two intestinal sampling sites. Differential abundance analysis with DESeq2 revealed that the mucosa is significantly enriched for the genus *Granulicatella* of the family *Carnobacteriaceae* (Figure 2E; Log2 fold change = 5.0, adjusted-*p* = 9.1E-6, Wald test followed by Benjamini–Hochberg correction for multiple testing), as well as for several typically mucosal-associated Firmicutes and Proteobacteria genera such as *Vibrio*, *Veillonella*, *Neisseria*, or *Haemophilus* (Figure 2E, Log2 fold change >2, adjusted-*p* < 0.05). Digesta communities are in turn enriched in many *Bacillaceae* genera, *Pseudomonas*, *Legionella*, as well as Actinobacteria genera such as *Corynebacterium*, *Mycobacterium*, *Brevibacterium*, or *Aeromicrobium* (Figure 2E, Log2 fold change <−2, adjusted-*p* < 0.05).

### Impact of Distinct IM on the European Sea Bass Gut Microbiota

**Figure 3:**
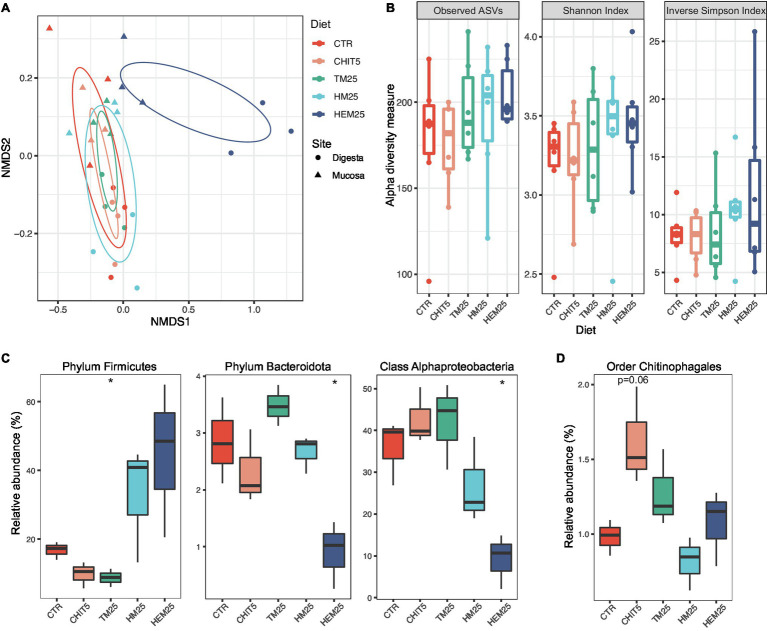
Insect meal-based diets induce major shifts in the European sea bass gut microbiome. **(A)** Ordination plot based on non-metric multidimensional scaling analysis of Bray–Curtis distances at the ASV level shown in Figure 2B, but now with individual data points colored by diet and shaped by sampling site. SD ellipses (colored by diet) are also depicted. **(B)** Alpha diversity metrics (number of Observed ASVs, Shannon Index, and Inverse Simpson Index) of samples from different diets. Each point represents one sample (*n* = 6 samples per diet). **(C,D)** Relative abundances of major taxa present in the fish digesta **(C)** or mucosa **(D)** which are significantly altered according to diet (*n* = 3). Phylum Firmicutes: ^*^p = 0.020; Phylum Bacteroidota: ^*^p = 0.027; Class Alphaproteobacteria: ^*^p = 0.011; Welch two-sample *t* test. In **(B–D)**, boxes represent median, first and third quartile. Whiskers extend to the highest and lowest values that are within one and a half times the interquartile range.

Beta-diversity analysis of samples from fish fed the experimental diets revealed an overall impact of diet on the composition of the European sea bass gut microbiota (*p* = 0.004, *r*^2^ = 0.244, PERMANOVA, Figure 3A). Among the tested diets, HI diets led to more pronounced shifts in microbial community composition, with major shifts detected in HEM25 (*p* = 0.041, *r*^2^ = 0.199, PERMANOVA; Supplementary Table 5). The shifts are accompanied by a slight increase in alpha diversity (Figure 3B), although this increase was not statistically significant. This is in line with other studies where IM-based diets were fed to the European sea bass (Antonopoulou et al., 2019; Pérez-Pascual et al., 2020; Panteli et al., 2021).

Because the sampling habitat (mucosa vs. digesta) strongly influences community composition, as shown above, the impact of the diets on each of the two sampled sites were also analyzed independently. We did not observe any diet-driven significant alterations in alpha diversity within samples coming from either site (Supplementary Figure 1). However, results show that the type of diet more strongly influences the digesta community than the mucosa community (Figure 3A; Supplementary Figure 1; Supplementary Table 5). Both HI-derived diets (HM25 and HEM25) lead to a digesta-specific increase in the relative abundance of the phylum Firmicutes, while the relative abundance of this phylum decreases in fish fed the TM diet (Figures 1D, 3C). In contrast, both the phylum Bacteroidota and the class Alphaproteobacteria are significantly decreased in relative abundance in fish fed the HEM25 diet (Figure 3C). In the mucosa, the only major shift at a high taxonomic level was detected for the order Chitinophagales in fish fed a diet supplemented with the commercial shrimp chitin (CHIT5; Figure 3D). At lower taxonomic levels no significant shifts were detected in fish fed a CHIT5 diet (adjusted-*p* < 0.05, DESeq2 analysis; Supplementary Table 6). Microbiota modulation by the mealworm-based diet (TM25) was modest and occurred only in the digesta, being characterized by an increase in the Betaproteobacteria genus *Cupriavidus* (from 0.3% to 2.3% increase in relative abundance), and by decreases in the family *Lactobacillaceae* and the genus *Ligilactobacillus*, which decreases from 11.2% to 4.9% in relative abundance (adjusted-*p* < 0.05, DESeq2 analysis; Supplementary Table 6).

**Figure 4:**
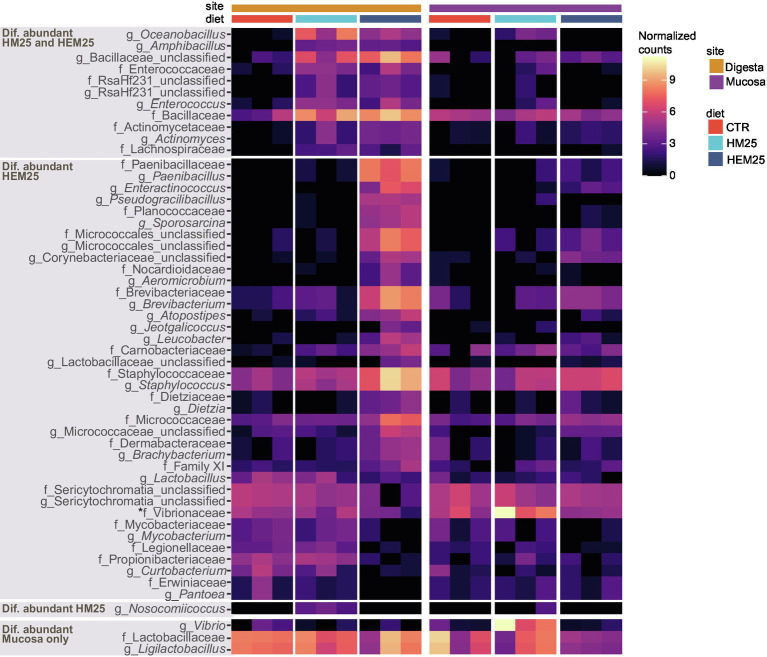
Genus and Family-level shifts induced by HI-based diets. Heat map showing transformed (log2 base) normalized read counts of genus and family-level taxa found to be differentially abundant in the guts of fish fed HM25 vs. a CTR diet, or fed a HME25 vs. CTR diet, based on DESeq2 analysis (adjusted value of *p* < 0.05, Wald test followed by Benjamini–Hochberg correction for multiple testing). Top three panels show taxa differentially abundant in digesta-originating samples or in both digesta and mucosa samples (marked with an asterisk), while the bottom panel shows taxa differentially abundant in mucosa-originating samples only.

To better understand the modulatory effect of HEM and HM, we proceeded to identify specific microbial families and genera that had altered abundances due to HEM or HM inclusion in the diets using DESeq2. A total of 50 taxa (22 families and 28 genera) and 12 taxa (five families and seven genera) were found to be differentially abundant in the digesta of fish fed the HEM25 or HM25 diets, respectively, when compared with the CTR diet (Figure 4; Supplementary Table 6: adjusted-*p* < 0.05, Wald test followed by Benjamini–Hochberg correction for multiple testing). Of these, the vast majority (40 out of 50 taxa for the HEM25 diet, and all 12 taxa of the HM25 diet) were shown to be more abundant in the HEM an HM diets compared with the CTR (Figure 4). These taxa largely belong to the phylum Firmicutes, and to a less extent to the phylum Actinobacteria, in line with the results presented above (Figures 1A, 2C). Taxa who increase in abundance on both diets correspond to the genera *Oceanibacillus*, *Amphibacillus* and unclassified *Bacillaceae* (family *Bacillaceae*, which sharply increases from 1.0% to 12.8% and 12.4% in relative abundance for HM25 and HEM25 diets, respectively), *Actinomyces* (*Actinomycetaceae*), *Enterococcus* (*Enterococcaceae*) and *Lachnospiraceae*. Consumption of HEM25 diet, but not of HM25, leads to a further increase in the Firmicutes genera *Paenibacillus* (*Paenibacillaceae*; from not detected to 5.5% in relative abundance) and *Staphylococcus* (*Staphylococcaceae*, from 1.1% to 14.8% in relative abundance), *Planococcaceae*, as well as in several Actinobacteria families such as *Brevibacteriaceae* and *Micrococcaceae* (an increase from 0.2% to 6.0%, and from 0.5% to 3.4% in relative abundance, respectively; Figure 4, panel “Dif. abundant HEM25”; Supplementary Tables 3, 6). In turn, organisms whose abundance decreases upon consumption of the HME25 diet belong predominantly to the class Alphaproteobacteria (*Legionellaceae*, *Vibrionaceae*, *Erwiniaceae*; Figures 2C, 4). Increase in the abundance of the *Staphylococcaceae* genus *Nosocomiicoccus* is only observed in fish fed the HM25 diet, but not the HEM25 diet (Figure 4; panel “Dif. abundant in HM25”). Shifts in abundance of many of these taxa were also detected for mucosal-associated communities and tended to follow the same trends as for the digesta communities (with few exceptions), although these were found not to be statistically significant (Figure 4; adjusted-*p* > 0.05, Wald test followed by Benjamini–Hochberg correction for multiple testing). The only taxa that significantly changed in abundance in the mucosa are the family *Vibrionaceae* and the *Vibrio* genus, which increases in abundance upon consumption of the HM25 diet, and the genus *Ligilactobacillus* (family *Lactobacillaceae*), which decreases upon consumption of the HEM25 diet (Figure 4, bottom panel).

### HI Exuviae Diet Drives an Increase in Abundance of Chitinase-Encoding *Paenibacillus* Species With Effective Chitinolytic Activity

**Figure 5:**
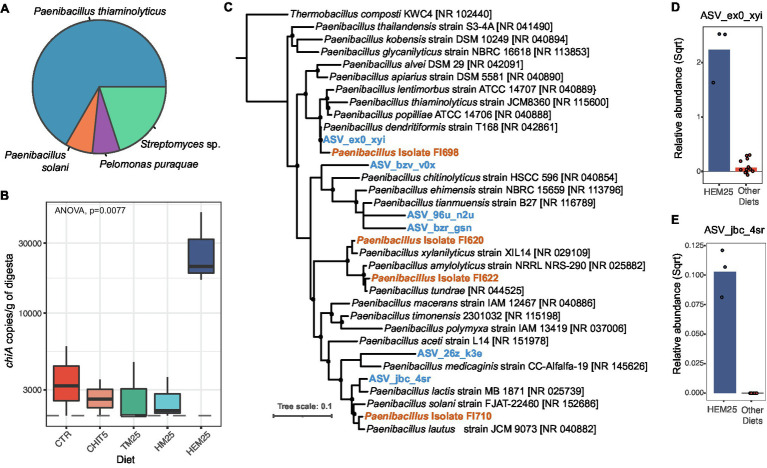
HI exuviae diet increases the abundance of Chitinase A-encoding taxa. **(A)** Taxonomic affiliation of cloned chitinase genes (*chi*A) amplified from digesta samples (*n* = 15 sequenced clones). **(B)** Copy number (determined by qPCR) of *chi*A in DNA samples obtained from the digesta of fish fed different diets. For some of the CHIT5, TM25 and HM25 diet samples, the presence of *chi*A was determined to be below the limit of detection of 10 copies per μl of DNA (equivalent to 2,000 copies per gram of digesta), represented by a dashed line. **(C)** Tree showing phylogenetic affiliations of ASVs and *Paenibacillus* sp. isolates with chitinolytic activity (recovered from the fish intestines) in relation to reference *Paenibacillus* isolate sequences. ASVs are presented in blue and bold, and isolates are highlighted in orange and bold. Bootstrap values >50% are presented as black filled circles on nodes. Scale bar represents 10% sequence divergence. **(D,E)** Relative abundance of ASV_eox_xvi **(D)** and ASV_jbc_4sr **(E)** (square root normalized) in digesta of fish fed HEM25 diet or all other diets combined. Bars represent mean values, and each point represents an individual sample (*n* = 3 for HEM25 diet and *n* = 12 for other diets).

Chitin degradation has been shown to enhance host and microbiota utilization of otherwise inaccessible proteins and lipids present in IM-based diets (De Marco et al., 2015). Genomic potential for chitin degradation and/or proven chitinolytic activity has been demonstrated for many bacteria of marine environments, including Gamma- and Beta-proteobacteria, Cyanobacteria, Bacteroidetes, Actinobacteria and Firmicutes (Ramaiah et al., 2000; Kawase et al., 2004; Hobel et al., 2005; Paulsen et al., 2016; Enke et al., 2018). Among the different reported types of bacterial chitinases (Orikoshi et al., 2005; Martínez-Zavala et al., 2020), ChiA has proven useful as a molecular marker for chitinase presence due to its high degree of sequence conservation and occurrence in a large array of phylogenetically distant organisms (Ramaiah et al., 2000; Hobel et al., 2005). To determine whether the dramatic shifts in microbial community structure and composition observed for the HM25 and HEM25 diets were accompanied by an increase in microbial chitinolytic potential, we screened samples for the presence of *chiA* using primers targeting a conserved region of the gene (Ramaiah et al., 2000). To confirm the selectivity of the primers, amplicon products were cloned and sequenced. All sequenced clones (*n* = 15) contained sequences matching chitinases. The vast majority (73%) of the chitinase sequences display high similarity (67.16%–98.78%) with chitinases originating from *Paenibacillus* sp. (*Paenibacillus thiaminolyticus* and *Paenibacillus solanis* or closely related organisms), followed by *Streptomyces* sp. (27%), with a single clone containing a sequence identical to a chitinase from the *Pelomonas puraquae* (Figure 5A). Using quantitative PCR we show that *chiA* is detected in the digesta of fish on all diets, but fish fed the HEM25 diet have significantly higher copy numbers of *chiA* compared to fish fed the CTR or any of the other diets (2.4 ± 0.2E04 vs. 3.7 ± 1.7E03 copies per gram of digesta; Figure 5B, *p* = 0.0077, ANOVA). Levels of *chiA* were below the limit of detection (10 copies per μl of DNA or 2E03 copies per gram of digesta) for all mucosal samples tested. These results show an increased *chiA*-driven chitinolytic potential of the European sea bass digesta microbiota fed a HEM25 diet.

Further, phylogenetic analysis of *Paenibacillus* 16S rRNA gene amplicon sequences revealed that two of the ASVs identified in our study (ASV_eox_xvi and ASV_jbc_4sr) are highly related to *P. thiaminolyticus* (100% identity) and *P. solanis* (97.2% identity), respectively (Figure 5C). Interestingly, both of these ASVs increased in relative abundance in fish fed the HEM25 diet (Figures 5D,E), thus potentially explaining the increased *chiA* levels associated with the HEM25 microbiota. To confirm that the increase in *chiA* copy number could be translated into an increase in effective microbiota-driven chitinolytic activity, we screened a collection of spore-forming organisms isolated from the guts of these fish for the presence of *Paenibacillus* isolates with chitinolytic capacity (Rangel et al., manuscript in preparation). All recovered *Paenibacillus* isolates displayed chitinolytic activity (Figure 5C, Isolates FI698, FI620, FI622, and FI710; see Materials and Methods). Based on phylogenetic analyses we confirm that two of these isolates (FI698 and FI710) are highly related (>98% identity) to the identified *Paenibacillus* ASVs mentioned above, and at least one of these isolates (FI698) contains a copy of *chiA*. Together, these results demonstrate that the HEM25 diet, but not any of the other IM diets, is able to modulate the fish digesta microbiota leading to an increase in the abundance of several chitinolytic *Paenibacillus* spp. and to overall microbiota-associated chitinolytic potential.

## Discussion

The rapid expansion of aquaculture dictates that, for a sustainable industry, aquafeeds must be adapted to the available resources. Changes in fish dietary regime, with the inclusion of novel ingredients such as IM, could however, reshape the microbial communities present in the digestive tract. As aquaculture turns to the inclusion of insect protein in fish diets, understanding microbial community responses to these ingredients is paramount. Several studies have evaluated the effects of IM-based diets on fish growth and digestibility (Nogales-Mérida et al., 2018), however, studies on its impact on the gut microbiota of marine fish are still in low number and in certain cases, inconclusive (Antonopoulou et al., 2019; Józefiak et al., 2019; Li et al., 2021; Panteli et al., 2021; Rimoldi et al., 2021; Terova et al., 2021; Tran et al., 2022). In this study, we unveil shifts occurring on European sea bass digesta- and mucosa-associated gut microbial communities in response to three different IM including, for the first time, an IM based on pupae exuviae.

Microbial composition analysis of the mucosa and digesta communities revealed high abundance of Proteobacteria, Firmicutes and Actinobacteria in the guts of the European sea bass in aquaculture, in agreement with previous reports (Kokou et al., 2019; Rimoldi et al., 2020; Serra et al., 2021). In this study we observed that mucosal communities are less diverse than digesta communities and are more resilient to changes induced by IM-based diets. These findings demonstrate that the intestinal sampling site acts as a filter, enabling the establishment of distinct microbial communities along the cross-sectional axis of the gut, similarly to what has been documented longitudinally along the different compartments of the European sea bass gastrointestinal tract (Kokou et al., 2019). The resilience of mucosa-associated communities of marine fish to dietary changes has been reported in other studies (Li et al., 2021; Serra et al., 2021), and is likely to be the result of stable symbiotic relationships established with the host and other microorganisms upon adhesion to the mucosal surface. Within the intestinal lumen, however, constant renovation of dietary nutrients and associated niches might allow the co-existence of otherwise competitive exclusive bacteria and, thus, greater variability (Pereira and Berry, 2017). Additionally, the lower richness and diversity of the autochthonous microbiota could also indicate that several allochthonous microorganisms do not possess the necessary characteristics to colonize the mucosal surface of the host (Gajardo et al., 2016; Serra et al., 2021).

Dietary components promoted pronounced digesta-associated microbial community shifts (Figure 3). When compared to the control diet, both HM25 and HEM25 lead to an increase in the relative abundance of the phylum Firmicutes in the digesta-associated microbial communities, whereas TM25 lead to a decrease. In fact, the increase of Firmicutes, generally thought to be advantageous due to the multiple beneficial bacteria known within this phylum (Perry et al., 2020; Van Doan et al., 2020; Ringø et al., 2020a,b), appears to be consistent independently of the fish species fed HM (Foysal and Gupta, 2022). At the family level, both HM diets lead to an increase of the families *Bacillaceae*, *Enterococcaceae*, and *Lachnospiraceae* (all Firmicutes), all of which contain species with beneficial potential: the *Bacillaceae* family is known to possess species with identified probiotic abilities such as the *Bacillus subtilis* (Butt and Volkoff, 2019; Van Doan et al., 2020; Ringø et al., 2020b); *Enterococcaceae* are known to produce antibacterial compounds and to display probiotic abilities, increasing the fish immunologic response and resistance to disease, as well as improving growth (Ringø et al., 2018, 2020a); and *Lachnospiraceae* has members capable of producing butyrate, a highly important short-chain fatty acid (SCFA), known to act as an anti-inflammatory agent and to play a key role in the fish immune system regulation (Terova et al., 2016; Zhang and Davies, 2016). Further, the HEM diet had an even stronger influence over the digesta-microbiota, increasing the abundance of additional families of the Firmicutes phylum such as *Planococcaceae*, *Staphylococcaceae*, and *Brevibacteriaceae*. Increases in these families due to dietary HM inclusion has also been shown by other authors (Terova et al., 2019; Panteli et al., 2021), although its function on the fish gut microbiota has been poorly discussed. Our data also revealed that HEM25 promoted an increase in *Paenibacillaceae* (Figure 4). This family, which was also shown to increase upon a dietary inclusion of 15% HM in rainbow trout (*Oncorhynchus mykiss*; Rimoldi et al., 2021), contains the genus *Paenibacillus*, known to have probiotic abilities when added to the fish feed (Gupta et al., 2014, 2016; Chen et al., 2019), as well as chitinolytic capabilities (discussed below).

Interestingly, we observed distinct changes in the gut microbiota community in response to the different diets used in this study, despite their similar crude protein, lipid, and chitin content (Figure 1). Inclusion of dietary fibers, such as chitin, generally leads to gut microbiota shifts toward Firmicutes and/or Actinobacteria (Huyben et al., 2019; Rimoldi et al., 2019; Terova et al., 2019; Panteli et al., 2021). However, similar dietary chitin contents (1.7% in HM25 vs. 1.8% in HEM25 vs. 1.3% in TM25) led to different levels in both Firmicutes (increased in HM25 and HEM25 vs. decrease in TM25), and Actinobacteria (increase in HM25 and HEM25 vs. no-effect in TM25), as well as to disparate shifts at low taxonomic levels (Figure 4). As with Firmicutes, selective increase of Actinobacteria in the guts of fish fed diets containing HM, appears to be transversal independently of the fish species (Foysal and Gupta, 2022). Moreover, dietary inclusion of 5% of commercial chitin did not result in the increase of bacteria of either of those phyla. This can be due to different physicochemical characteristics of chitin present in the different diets. Varying degrees of acetylation, chitin-associated proteins and insects’ life cycle stages can change, among others, chitin’s surface, porosity, or solubility thus affecting its bioaccessibility (Zargar et al., 2015; Waśko et al., 2016; Muthukrishnan et al., 2019; Purkayastha and Sarkar, 2020). Furthermore, we also hypothesize that dietary components other than chitin are behind some of the modulatory effects observed. Indeed, the constitution of macromolecules such as lipids and proteins differ according to the insect’s species, developmental stage and rearing substrate, which can result in different modulatory effects of the microbiota (Gołębiowski et al., 2014; Nogales-Mérida et al., 2018; Vogel et al., 2018; Bruno et al., 2019). As an example, fatty acid profile in lipids has been shown to differently impact gut microbial communities, with diets supplemented with medium chain fatty acids (MCFA) favoring an increase of the Firmicutes phylum (Rimoldi et al., 2018). The latter might help to explain the observed increase in the Firmicutes phylum in HM-based diets as HM, in contrast with TM, are rich in the MCFA lauric acid (C12:0; Guerreiro et al., 2020). Thus, diet-induced alterations in the gut microbiota may occur due to IM-specific fatty acid, amino acid, or even simple-sugar profiles, potentially creating micro-niches that promote the establishment of organisms with selective nutritional preferences (Pereira and Berry, 2017). This could also help explain the absence of microbiota shifts in response to a CHIT5 diet, which shares the same overall crude macromolecular composition as the control diet, except for the presence of chitin (Figure 1). Finally, we cannot exclude that some of the differences among diets originate from amplification of DNA from dead vegetative cells or from spores that were present in the own insect’s gut or cuticle. Other approaches such as transcriptomics or activity-based methods would be necessary to fully unravel the contribution of food-borne microorganisms to the overall fish gut microbiota profiles (Emerson et al., 2017).

The presence of chitin is a negative aspect of IM because it is difficult to breakdown and because it can entrap other nutrients, like lipids and proteins, thereby limiting their digestibility (De Marco et al., 2015; Guerreiro et al., 2021). Upon HM inclusion in diets, we observed an increase in Firmicutes families *Bacillaceae*, *Enterococcaceae*, and *Actinomycetaceae*, and a HEM-specific increase in the families *Planococcaceae*, *Brevibacteriaceae*, and *Paenibacillaceae*, all of which contain species with chitinolytic activity (Callegari et al., 2020; Shimoi et al., 2020). Indeed, we detected an increase in the copy number of genes encoding ChiA, used here as a marker for chitinolytic potential, in fish fed the HEM25 diet (Figure 5). However, we detected no increase in *chiA* copies in fish fed the CHIT5 diet, despite an increase of the order Chitinophagales which contains species that can degrade chitin (Rosenberg, 2014; Wieczorek et al., 2019), nor in fish fed the HM25 diet, despite an increase in *Bacillaceae* and *Actinomycetaceae* (Shimoi et al., 2020). Chitinolytic *Chitinopagaceae* species, along with Actinobacteria species, have been shown to employ chitinases other than ChiA, such as exochitinases or endochitinases belonging to other glycoside hydrolase families, for chitin breakdown (Lacombe-Harvey et al., 2018; Ramakrishna et al., 2018). As these chitinases share no sequence similarity with ChiA (Lacombe-Harvey et al., 2018), they may have been missed in our study. Apart from DNA-based evidence, future investigation of chitin-degradation capacity should include evaluation of the effective chitinolytic enzymatic activity of the digesta. Together, increases in abundance of 16S rRNA and *chiA* gene sequences belonging to *Paenibacillus* species in HEM25 diet, as well as recovery of *Paenibacillus* isolates with chitinolytic activity, support an HEM25-driven increase in chitinolytic potential that is likely translated into chitin degradation in the fish lumen. A detailed evaluation of the possible impact of HEM on fish fitness, at the inclusion levels here used or at higher inclusion levels, should be performed in the future.

## Conclusion

In summary, the data reported here provides a detailed characterization of microbiota shifts induced by two commonly used IM (TM and HM) and one underexplored insect-based ingredient (HEM) on the European sea bass gut microbiota. Overall, the data suggests that IM diets of different origins differently impact the gut microbiota of the European sea bass digesta-associated communities, while mucosal-associated communities are more resilient to diet-induced alterations. The inclusion of HEM in the diet increased the digesta bacterial-associated chitinolytic potential, likely resulting in enhanced utilization of otherwise inaccessible proteins, lipids, and energy present in this diet, which ultimately can lead to better fish growth performance. Our study uncovers possible advantages of incorporating exuviae in fish diets, an economically attractive product that may be key to raise levels of IM inclusion in aquafeeds.

## Data Availability Statement

The 16S rRNA gene sequences were deposited in the NCBI Sequence Read Archive (SRA) as BioProject Accession PRJNA767204. The datasets generated and/or analyzed during the current study are available in the github repository, under the link https://github.com/fatimapereira454/Amplicon-sequencing_Seabass_IMdiets (metadata table, read count table).

## Ethics Statement

The animal study was reviewed and approved by Animal Welfare Committee of the Interdisciplinary Centre of Marine and Environmental Research (CIIMAR) and carried out in a registered installation (N16091.UDER). The trial was performed by trained scientists (following FELASA category C recommendations) in full compliance with national rules and following the European Directive 2010/63/EU of the European Parliament and the European Union Council on the protection of animals used for scientific purposes.

## Author Contributions

CRS, PE, FCP, and AO-T designed and conceived the study. FR and FCP performed the experiments. FCP and BH generated and analyzed the sequencing data. LG and FG formulated and provided the diets. FR, FCP, CRS, and PE wrote the manuscript with contributions from AO-T and DB. All authors contributed to the article and approved the submitted version.

## Funding

This work was funded by the structured program of R&D&I ATLANTIDA—Platform for the monitoring of the North Atlantic Ocean and tools for the sustainable exploitation of the marine resources (NORTE-01-0145-FEDER-000040), supported by the North Portugal Regional Operational Programme (NORTE2020), through the European Regional Development Fund (ERDF), and by the Strategic Funding to UID/Multi/04423/2019 (POCI-01-0145-FEDER-007621), through national funds provided by the Portuguese funding agency for science, research and technology (FCT) and European Regional Development Fund (ERDF), in the framework of the programme PT2020. FR was supported by a grant from FCT, Portugal (SFRH/BD/138375/2018). CRS and PE have a scientific employment contract supported by national funds through FCT. This research was also funded by the Austrian Science Fund (FWF) *via* a Young Independent Research Group grant (ZK-57) to FCP.

## Conflict of Interest

The authors declare that the research was conducted in the absence of any commercial or financial relationships that could be construed as a potential conflict of interest.

## Publisher’s Note

All claims expressed in this article are solely those of the authors and do not necessarily represent those of their affiliated organizations, or those of the publisher, the editors and the reviewers. Any product that may be evaluated in this article, or claim that may be made by its manufacturer, is not guaranteed or endorsed by the publisher.

## Footnotes

^1^ https://sourceforge.net/projects/bbmap/

^2^ https://github.com/jfjlaros/demultiplex

^3^ https://www.r-project.org/

^4^ https://www.ncbi.nlm.nih.gov/
