# Supplementary material for: Differential Modulation of the European Sea Bass Gut Microbiota by Distinct Insect Meals
Source: Front Microbiol. 2022 Apr 12;13:831034. doi: 10.3389/fmicb.2022.831034 (PMC9041418; doi:10.3389/fmicb.2022.831034)
Supplement: Supplementary file 1 [file Data_Sheet_1.PDF]

Supplementary Material

Differential modulation of the European sea bass gut microbiota by insect meals of distinct origins

F. Rangel[1,2], P. Enes[1,2] , L. Gasco[3], F. Gai[4], B. Hausmann[5,6], D. Berry[5,7], A. Oliva Teles[1,2], C. R. Serra[1,2,*] and F. C. Pereira[7]

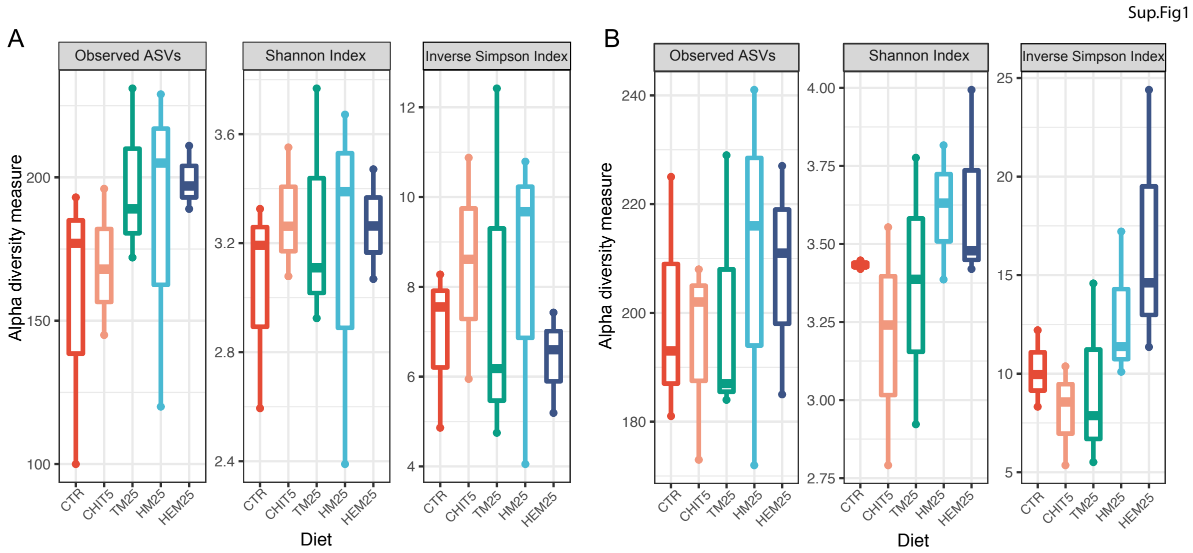

Supplementary Figure 1. Alpha diversity metrics (number of Observed ASVs, Shannon Index and Inverse Simpson Index) of the mucosa (**A**) and digesta samples (**B**) from fish fed indicated diets. Each point represents one sample (*n*=3 samples per diet per site; each sample was obtained after pooling material from 2 fish from the same tank). Boxes represent median, first and third quartile. Whiskers extend to the highest and lowest values that are within one and a half times the interquartile range. No significant diferences between diets were detected (p>0.05, Wilcoxon test).

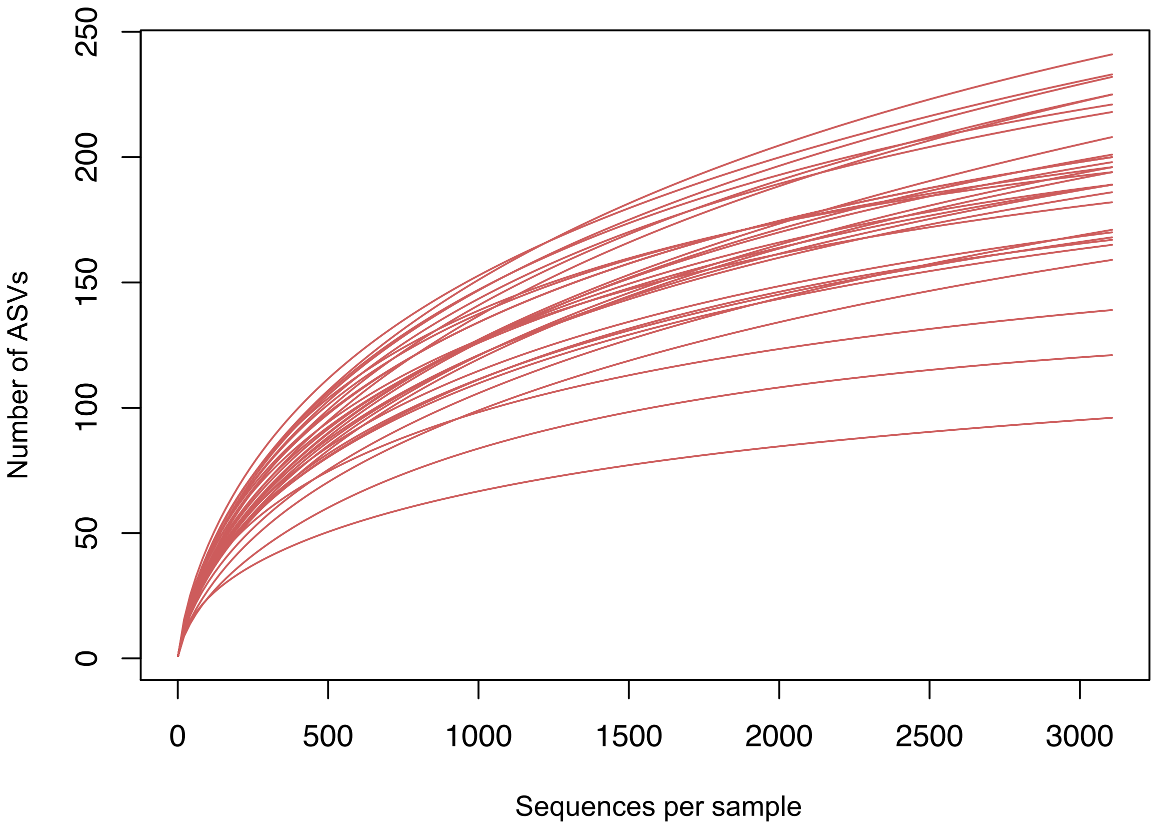

Supplementary Figure 2. Rarefaction curves for the different samples, after rarefying.

Supplementary Table 1. Ingredient composition and proximate analysis of the experimental diets.

	Diets				
	**CTR**	**HM25**	**HEM25**	**TM25**	**CHIT5**
Ingredients (% dry weight basis)					
Fish meal[a]	44.0	24.4	21.6	19.7	45.1
Soluble fish protein concentrate[b]	2.0	2.0	2.0	2.0	2.0
Hermetia illucens larvae meal[c]	-	25.0	-	-	-
Hermetia illucens exuviea meal[d]	-	-	25.0	-	-
Tenebrio molitor larvae meal[e]	-	-	-	25.0	-
Corn gluten[f]	5.0	5.0	5.0	5.0	5.0
Soybean meal[g]	7.5	7.5	7.5	7.5	7.5
Wheat meal[h]	27.9	20.4	22.1	25.9	21.9
Fish oil	9.9	11.7	11.7	10.5	9.7
Vitamin premix[i]	1.0	1.0	1.0	1.0	1.0
Mineral premix[j]	1.0	1.0	1.0	1.0	1.0
Choline chloride (50%)	0.5	0.5	0.5	0.5	0.5
Binder[k]	1.0	1.0	1.0	1.0	1.0
Taurine[l]	0.2	0.2	0.2	0.2	0.2
Dibasic calcium phosphate	-	0.3	1.4	0.7	-
Chitin[m]	-	-	-	-	5.0
Proximate analyses (% dry weight basis)					
Dry matter	93.6	92.8	89.7	93.4	93.6
Crude protein	46.0	45.4	45.2	45.3	46.3
Crude fat	17.8	17.9	18.1	17.8	18.2
Ash	7.8	8.3	8.6	6.7	7.7
Chitin	-	1.7	1.8	1.3	5.0

[a]Sorgal, S.A. Ovar, Portugal (CP: 72.4 % DM; GL: 17.0 %DM).
[b]Sorgal, S.A. Ovar, Portugal (CP: 79.7 % DM; GL: 7.14 % DM).
[c]Black soldier fly larvae meal (CP: 55.4 % DM; GL: 10.9 % DM; chitin 6.8 % DM).
[d]Black soldier fly exuviea meal (CP: 64.3 % DM; GL: 8.1 % DM; chitin 7.2 % DM).
[e]Yellow mealworm larvae meal (CP: 69.5 % DM; GL: 14.1 % DM; chitin 5.2 % DM).
[f]Sorgal, S.A. Ovar, Portugal (CP: 69.9 % DM; GL: 3.3 % DM).
[g]Sorgal, S.A. Ovar, Portugal (CP: 54.2 % DM; GL: 1.8% DM).
[h]Sorgal, S.A. Ovar, Portugal (CP: 13.8 % DM; GL: 1.1 % DM).
[i]Vitamins (mg kg^{-1} diet): retinol, 18 000 (IU kg^{-1} diet); cholecalciferol, 2 000 (IU kg^{-1} diet); α- tocopherol, 35; menadione sodium bisulphate, 10; thiamine, 15; riboflavin, 25; Ca pantothenate, 50; nicotinic acid, 200; pyridoxine, 5; folic acid, 10; cyanocobalamin, 0.02; biotin, 1.5; ascorbyl monophosphate, 50; inositol, 400.
[j]Minerals (mg kg^{-1} diet): cobalt sulphate, 1.91; copper sulphate, 19.6; iron sulphate, 200; sodium fluoride, 2.21; potassium iodide, 0.78; magnesium oxide, 830; manganese oxide, 26; sodium selenite, 0.66; zinc oxide, 37.5; dibasic calcium phosphate, 5.93 (g kg^{-1} diet); potassium chloride, 1.15 (g kg^{-1} diet); sodium chloride, 0.44 (g kg^{-1} diet).
[k]Aquacube. Agil, UK.
[l]Feed-grade taurine, Sorgal, S.A. Ovar, Portugal.
[m]Chitin from shrimp shells, practical grade powder, Sigma-Aldrich

Supplementary Table 2. Coverage (as a measure of sample completeness) for each analysed sample.

Sample ID	Sample type	Diet	Sample Coverage
D1	Digesta	CTR	0.98
D2	Digesta	TM25	0.98
D3	Digesta	HEM25	0.97
D4	Digesta	HM25	0.98
D5	Digesta	TM25	0.98
D6	Digesta	CHIT5	0.98
D7	Digesta	CTR	0.98
D8	Digesta	CHIT5	0.98
D9	Digesta	HEM25	0.98
D10	Digesta	CHIT5	0.98
D11	Digesta	HM25	0.98
D12	Digesta	CTR	0.98
D13	Digesta	HEM25	0.98
D14	Digesta	TM25	0.98
D15	Digesta	HM25	0.98
M1	Mucosa	CTR	0.98
M2	Mucosa	TM25	0.98
M3	Mucosa	HEM25	0.98
M4	Mucosa	HM25	0.99
M5	Mucosa	TM25	0.98
M6	Mucosa	CHIT5	0.99
M7	Mucosa	CTR	0.99
M8	Mucosa	CHIT5	0.99
M9	Mucosa	HEM25	0.98
M10	Mucosa	CHIT5	0.98
M11	Mucosa	HM25	0.98
M12	Mucosa	CTR	0.98
M13	Mucosa	HEM25	0.98
M14	Mucosa	TM25	0.98
M15	Mucosa	HM25	0.98

Supplementary Table 3. Relative abundances of all taxa (Kingdom, Phylum, Class, Order, Family and Genus level) *(Attached Excel File)*.

Supplementary Table 4. Alpha diversity metrics.

Sample_ID	tank	site	diet	Observed ASVs	Shannon	InvSimpson
Digesta samples						
D1	1	Digesta	CTR	193	3.45	12.21
D7	7	Digesta	CTR	177	3.40	9.83
D12	12	Digesta	CTR	217	3.37	8.38
D3	3	Digesta	HE25	211	3.48	12.24
D9	9	Digesta	HE25	198	3.55	14.65
D13	13	Digesta	HE25	225	4.03	25.46
D2	2	Digesta	TM25	232	3.78	14.88
D5	5	Digesta	TM25	173	2.97	5.91
D14	14	Digesta	TM25	183	3.32	7.48
D4	4	Digesta	HM25	208	3.74	15.94
D11	11	Digesta	HM25	241	3.63	10.10
D15	15	Digesta	HM25	170	3.36	10.83
D6	6	Digesta	CHT	197	3.56	10.10
D8	8	Digesta	CHT	163	2.75	5.09
D10	10	Digesta	CHT	200	3.22	8.35
Mucosal samples						
M1	1	Mucosa	CTR	177	3.19	8.27
M7	7	Mucosa	CTR	92	2.52	4.70
M12	12	Mucosa	CTR	192	3.32	7.52
M3	3	Mucosa	HE25	194	3.50	7.72
M9	9	Mucosa	HE25	190	3.06	5.21
M13	13	Mucosa	HE25	231	3.29	6.57
M2	2	Mucosa	TM25	229	3.73	12.16
M5	5	Mucosa	TM25	162	3.08	6.09
M14	14	Mucosa	TM25	184	2.91	4.66
M4	4	Mucosa	HM25	117	2.39	4.17
M11	11	Mucosa	HM25	229	3.67	10.79
M15	15	Mucosa	HM25	203	3.39	9.92
M6	6	Mucosa	CHT	152	3.28	8.34
M8	8	Mucosa	CHT	166	3.08	6.01
M10	10	Mucosa	CHT	202	3.59	10.69

Supplementary Table 5. PERMANOVA analysis.

adonis[a]	Digesta and mucosa		Digesta		Mucosa	
	p-value	R^2	p-value	R^2	p-value	R^2
All diets	0.004	0.247	0.002	0.580	0.396	0.291
CHIT5 vs CTR	0.682	0.070	0.700	0.161	1.000	0.128
TM25 vs CTR	0.548	0.079	0.300	0.234	1.000	0.078
HM25 vs CTR	0.378	0.094	0.200	0.259	0.900	0.163
HEM25 vs CTR	0.041	0.199	0.100	0.567	0.600	0.181

[a]adonis() function in R for analysis of variance using distance matrices.

Supplementary Table 6. DESeq2 analysis of differentially abundant Genera and Families for the tested diets TM25, HM25 and HEM25 versus control (CTR) diet, for either digesta (TM25) or digesta and mucosal communities (HM25 and HEM25). For diet CHIT5 no significant differences were detected.

**Taxa[a]**	**base Mean[b]**	**log2_Fold Change**	**lfcSE[c]**	**p-value**	**adjusted p-value**
TM25 versus CTR-Digesta					
g_Cupriavidus	41.9694	2.5804	0.6260	0.0000	0.0103
f_Lactobacillaceae	291.0094	-1.1312	0.3028	0.0002	0.0259
g_Ligilactobacillus	247.2979	-1.1327	0.3152	0.0003	0.0301
HEM25 versus CTR-Digesta					
f_Paenibacillaceae	109.5574	10.6415	1.4788	0.0000	0.0000
g_Paenibacillus	107.2806	10.6112	1.4780	0.0000	0.0000
f_Brevibacteriaceae	135.0298	6.4369	1.0134	0.0000	0.0000
g_Brevibacterium	135.0298	6.4369	1.0134	0.0000	0.0000
g_Bacillaceae_unclassified	190.0325	6.9860	1.1096	0.0000	0.0000
g_Enteractinococcus	42.7933	9.2848	1.4941	0.0000	0.0000
f_Micrococcales_unclassified	82.5225	7.6867	1.2744	0.0000	0.0000
g_Micrococcales_unclassified	82.5225	7.6867	1.2744	0.0000	0.0000
g_Pseudogracilibacillus	18.5842	8.0806	1.3574	0.0000	0.0000
f_Planococcaceae	18.0009	8.0390	1.3671	0.0000	0.0000
g_Sporosarcina	16.9006	7.9483	1.3699	0.0000	0.0000
f_Carnobacteriaceae	17.0636	5.5299	1.1101	0.0000	0.0000
g_Oceanobacillus	14.1811	6.7114	1.3700	0.0000	0.0000
g_Atopostipes	16.7539	6.0937	1.2661	0.0000	0.0000
f_Staphylococcaceae	323.8769	4.5873	0.9609	0.0000	0.0000
g_Staphylococcus	320.0448	4.5694	0.9598	0.0000	0.0000
g_Corynebacteriaceae_unclassified	13.6506	6.6594	1.4110	0.0000	0.0000
g_Leucobacter	14.6242	5.7962	1.3043	0.0000	0.0001
f_Bacillaceae	278.0321	4.5757	1.0328	0.0000	0.0001
f_Propionibacteriaceae	16.4044	-4.5670	1.0716	0.0000	0.0002
f_Nocardioidaceae	6.3181	6.5159	1.5800	0.0000	0.0003
g_Aeromicrobium	6.3181	6.5159	1.5800	0.0000	0.0003
g_Curtobacterium	13.7607	-4.9123	1.2760	0.0001	0.0009
f_Micrococcaceae	69.6549	3.5101	0.9400	0.0002	0.0013
f_Actinomycetaceae	6.1038	5.4803	1.5026	0.0003	0.0017
g_Actinomyces	6.1038	5.4803	1.5026	0.0003	0.0017
f_Enterococcaceae	10.4342	3.9177	1.1038	0.0004	0.0023
f_RsaHf231_unclassified	4.0979	5.8955	1.6662	0.0004	0.0023
g_Enterococcus	10.4342	3.9177	1.1038	0.0004	0.0023
g_RsaHf231_unclassified	4.0979	5.8955	1.6662	0.0004	0.0023
f_Dermabacteraceae	11.5417	3.3097	0.9632	0.0006	0.0029

g_Amphibacillus	3.8502	5.8066	1.6905	0.0006	0.0029
g_Brachybacterium	11.5417	3.3097	0.9632	0.0006	0.0029
g_Micrococcaceae_unclassified	22.2801	3.0326	0.8827	0.0006	0.0029
f_Dietziaceae	7.4570	3.8792	1.1541	0.0008	0.0036
g_Dietzia	7.4570	3.8792	1.1541	0.0008	0.0036
f_Sericytochromatia_unclassified	28.9179	-2.8432	0.8520	0.0008	0.0038
g_Sericytochromatia_unclassified	28.9179	-2.8432	0.8520	0.0008	0.0038
f_Demequinaceae	3.6187	5.7296	1.7784	0.0013	0.0054
g_Demequinaceae_unclassified	3.6187	5.7296	1.7784	0.0013	0.0054
g_Lactobacillus	15.2746	-2.7128	0.8615	0.0016	0.0068
f_Erwiniaceae	4.8241	-5.2970	1.7485	0.0025	0.0096
g_Pantoea	4.8241	-5.2970	1.7485	0.0025	0.0096
f_Legionellaceae	5.6015	-4.4736	1.5195	0.0032	0.0124
f_Family XI	10.8410	2.4355	0.8825	0.0058	0.0217
f_Lachnospiraceae	2.5335	5.2174	1.9346	0.0070	0.0257
g_Lactobacillaceae_unclassified	3.9555	4.8413	1.8042	0.0073	0.0262
f_Mycobacteriaceae	5.9562	-3.5990	1.3971	0.0100	0.0345
g_Mycobacterium	5.9562	-3.5990	1.3971	0.0100	0.0345
g_Family XI_unclassified	3.2372	4.5181	1.8004	0.0121	0.0409
HM25 versus CTR-Digesta					
g_Oceanobacillus	65.6323	9.2829	1.4807	0.0000	0.0000
g_Amphibacillus	6.8354	6.4464	1.3973	0.0000	0.0004
g_Bacillaceae_unclassified	174.5504	4.9680	1.1015	0.0000	0.0005
f_Enterococcaceae	15.3439	4.1707	0.9978	0.0000	0.0010
f_RsaHf231_unclassified	6.8103	6.3838	1.5353	0.0000	0.0010
g_Enterococcus	15.3439	4.1707	0.9978	0.0000	0.0010
g_RsaHf231_unclassified	6.8103	6.3838	1.5353	0.0000	0.0010
f_Bacillaceae	330.9931	4.0901	0.9914	0.0000	0.0010
f_Actinomycetaceae	7.8338	5.2402	1.5037	0.0005	0.0107
f_Lachnospiraceae	3.9602	5.5091	1.5913	0.0005	0.0107
g_Actinomyces	7.8338	5.2402	1.5037	0.0005	0.0107
g_Nosocomiicoccus	2.8863	5.9964	1.8674	0.0013	0.0242
HEM25 versus CTR-Mucosa					
f_Lactobacillaceae	240.3031	-3.9755	0.9790	0.0000	0.0067
g_Ligilactobacillus	187.3716	-3.9730	0.9658	0.0000	0.0067
HM25 versus CTR-Mucosa					
g_Vibrio	412.4551	7.5674	1.2042	0.0000	0.0000
f_Vibrionaceae	457.5447	3.9646	1.0089	0.0001	0.0113

[a] The prefix "f_" denotes family and "g_" denotes genus.
[b] Base count mean.
[c] Standard error value returned by DESeq2.

Supplementary Table 6. DESeq2 analysis of differentially abundant Genera and Families in diet HEM25 versus control and H;25 versus control, for both digesta and mucosal communities.

clone ID[a]	BLAST NCBI[b]			
	**Identity**	**Sequence ID**	**Identity %**	**coverage**
ChiA_A1	chitinase [Paenibacillus solani]	WP_054405196.1	67.24	94
ChiA_A2	hypothetical protein [Pelomonas puraquae] (GH18)	WP_158218444.1	98.15	98
ChiA_A3	chitinase [Paenibacillus thiaminolyticus]	WP_143798588.1	98.72	97
ChiA_A4	family 18 chitinase, Partial [Streptomyces coelicoflavus]	SNU02820.1	67.16	98
ChiA_A5	chitinase [Paenibacillus thiaminolyticus]	WP_119791674.1	97.26	98
ChiA_B1	chitinase [Paenibacillus thiaminolyticus]	WP_119791674.1	98.78	98
**ChiA_B2**	**chitinase [Paenibacillus thiaminolyticus]**	**WP_119791674.1**	**98.78**	**98**
ChiA_B3	chitinase [Paenibacillus thiaminolyticus]	WP_119791674.1	96.72	98
ChiA_B4	chitinase [Paenibacillus thiaminolyticus]	WP_119791674.1	98.63	84
ChiA_B5	chitinase [Paenibacillus thiaminolyticus]	WP_143798588.1	98.63	84
ChiA_B6	family 18 chitinase, Partial [Streptomyces lasiicapitis]	SNU02819.1	68.75	94
ChiA_B7	MULTISPECIES: chitinase [Streptomyces]	WP_030882014.1	71.64	98
ChiA_C1	chitinase [Paenibacillus thiaminolyticus]	WP_087440603.1	98.18	71
ChiA_C2	chitinase [Paenibacillus thiaminolyticus]	WP_143798588.1	98.78	98
ChiA_C3	chitinase [Paenibacillus thiaminolyticus]	WP_119791674.1	96.72	97

[a] Clone highlighted in bold (ChiA_B2) was used as template for qPCR standards.
[b] https://blast.ncbi.nlm.nih.gov/ (Blastx option was used).
